# Circulating Level of Growth‐Differentiation Factor 15 and Mortality of Patients With Acute Heart Failure: A Meta‐Analysis

**DOI:** 10.1002/clc.70338

**Published:** 2026-05-06

**Authors:** Pingkui Jin, Yanjie Geng, Erwei Huo, Yanhong Xue, Daofeng You, Qinghou Zheng

**Affiliations:** ^1^ Department of Emergency The First Hospital of Hebei Medical University Shijiazhuang China; ^2^ Department of Cardiology The First Hospital of Hebei Medical University Shijiazhuang China

**Keywords:** acute heart failure, growth‐differentiation factor 15, meta‐analysis, mortality, risk factor

## Abstract

**Background:**

Growth differentiation factor‐15 (GDF‐15) is a stress‐responsive biomarker implicated in inflammation and myocardial injury. Its prognostic value for mortality risk in acute heart failure (AHF) remains uncertain. This meta‐analysis evaluated the association between elevated admission circulating GDF‐15 levels and subsequent mortality in patients hospitalized with AHF.

**Methods:**

PubMed, Embase, and Web of Science were systematically searched for prospective or retrospective cohort studies and post‐hoc trial analyses enrolling adult AHF patients with blood GDF‐15 measured on admission. Risk ratios (RRs) for all‐cause mortality comparing high versus low GDF‐15 categories were pooled using random‐effects models incorporating the influence of potential heterogeneity.

**Results:**

Ten studies with 3724 patients with AHF were included. Overall, high admission GDF‐15 levels were significantly associated with increased mortality risk during follow‐up (RR = 2.82, 95% CI: 2.39–3.32; *p* < 0.001), with no evidence of between‐study inconsistency (*I*² = 0%). Sensitivity analyses confirmed robustness (leave‐one‐out RR range: 2.73–3.00), and results remained consistent in high‐quality studies (NOS ≥ 8; RR = 2.72, 95% CI: 2.26–3.27). Subgroup analyses demonstrated similar associations across Asian and Western cohorts, prospective and retrospective designs, different sampling times (at admission to within 48 h), assay methods (ELISA vs. ECLIA), cutoff definitions, follow‐up duration, and adjustment for BNP/NT‐proBNP (all *p* for subgroup differences >0.05). No significant publication bias was detected (Egger's *p* = 0.59).

**Conclusions:**

Elevated circulating GDF‐15 levels at admission are strongly associated with increased mortality risk in patients with AHF, supporting its potential role in early risk stratification.

## Graphical abstract

Elevated admission GDF‐15 levels are associated with increased mortality in acute heart failure. This meta‐analysis of 10 studies (*N* = 3724) found a pooled RR of 2.82 (95% CI: 2.39–3.32; *p* < 0.001; *I*² = 0%), supporting GDF‐15 as a promising biomarker for early risk stratification in AHF patients.

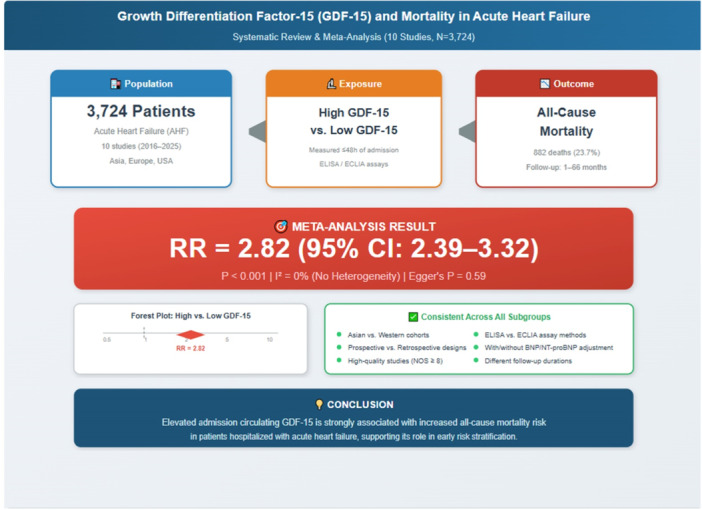

## 1 Introduction

Acute heart failure (AHF) is a life‐threatening clinical syndrome characterized by the rapid onset or worsening of heart failure (HF) symptoms requiring urgent hospitalization [1, 2]. It represents one of the leading causes of emergency admissions among older adults worldwide and imposes a substantial burden on healthcare systems through high rates of in‐hospital complications, early readmissions, and long‐term mortality [3, 4]. Despite advances in evidence‐based therapies and supportive care, patients hospitalized with AHF remain at substantial risk of adverse outcomes, including early mortality and rehospitalization [5]. However, prognosis is highly heterogeneous across individuals, and accurately identifying those at greatest risk remains a clinical challenge [6]. Accordingly, improved risk stratification at the time of admission is essential to guide clinical decision‐making, optimize monitoring intensity, and enable more personalized management strategies [7, 8]. While natriuretic peptides and cardiac troponins are widely used in routine practice, these biomarkers primarily reflect hemodynamic stress or myocardial injury and do not fully capture the complex systemic processes underlying adverse outcomes in AHF [9]. Accordingly, there is an ongoing need to identify novel prognostic biomarkers that integrate inflammatory, metabolic, and multi‐organ stress pathways to enhance early mortality prediction and refine clinical risk assessment [10].

Growth differentiation factor‐15 (GDF‐15), also known as macrophage inhibitory cytokine‐1, is a member of the transforming growth factor‐β superfamily that is upregulated in response to cellular stress, inflammation, oxidative injury, and tissue hypoxia [11, 12]. GDF‐15 is expressed in multiple organs, including the myocardium, vascular endothelium, and kidneys, and has emerged as an integrative biomarker reflecting systemic disease severity beyond traditional cardiac‐specific markers [13]. In cardiovascular medicine, elevated circulating GDF‐15 levels have been associated with adverse outcomes in chronic HF [14], acute coronary syndromes [15], and cardiometabolic disorders [16], highlighting its potential utility for prognostic enrichment and therapeutic risk targeting. Mechanistically, increased GDF‐15 in AHF may reflect heightened inflammatory activation, endothelial dysfunction, renal impairment, and progressive myocardial remodeling, thereby linking acute systemic stress responses to increased mortality risk [17]. Previous studies have explored the prognostic value of GDF‐15 in various HF settings. Meta‐analyses in patients with chronic and stable HF have demonstrated that elevated circulating GDF‐15 levels are associated with an increased risk of mortality and adverse outcomes [18]. In addition, recent evidence suggests that GDF‐15 may provide incremental prognostic value across different HF phenotypes, including HF with preserved, mildly reduced, and reduced ejection fraction [19]. However, AHF differs from chronic disease by its rapid hemodynamic deterioration and dynamic systemic changes, meaning biomarkers measured at this stage may reflect distinct pathophysiology and prognostic information [20]. Although several cohort studies have examined the association between admission GDF‐15 levels and mortality outcomes in AHF [21, 22, 23, 24, 25, 26, 27, 28, 29, 30], findings have been reported across heterogeneous populations, assay methods, cutoff definitions, and follow‐up durations, limiting the clinical interpretability of individual studies. Therefore, the present systematic review and meta‐analysis aimed to comprehensively synthesize available evidence on the prognostic value of elevated admission circulating GDF‐15 levels for predicting all‐cause mortality in patients hospitalized with AHF, and to explore the consistency of this association across key study characteristics.

## 2 Methods

This meta‐analysis was conducted and reported in accordance with the PRISMA 2020 guidelines [31] and the Cochrane Handbook [32], covering protocol development, data collection, statistical synthesis, and reporting of results. The study protocol was prospectively registered in PROSPERO (CRD420261307047).

### 2.1 Database Search

Eligible studies were identified through a comprehensive and systematic literature search of PubMed, Embase, and Web of Science. A broad set of predefined search terms was applied, incorporating keywords and synonyms related to: (1) growth differentiation factor‐15, including “growth differentiation factor 15,” “macrophage inhibitory cytokine 1,” “prostate differentiation factor,” “GDF‐15,” “GDF 15,” and “MIC‐1”; (2) heart failure, including “heart failure,” “cardiac failure,” “cardiac dysfunction,” and “cardiac insufficiency”; (3) the acute clinical setting, using terms such as “acute,” “acutely,” “decompensate,” “decompensation,” and “decompensated”; and (4) mortality and prognostic outcomes, including “mortality,” “death,” “survival,” “outcome,” “prognosis,” “prognostic,” as well as terms reflecting longitudinal follow‐up and cohort study designs (e.g., “cohort,” “prospective,” “retrospective,” and “follow‐up”). The search was restricted to studies conducted in humans and to full‐text articles published in English in peer‐reviewed journals. To ensure completeness, the reference lists of relevant original articles and review papers were also manually screened to identify additional eligible publications not captured through the electronic search. All databases were searched from their inception through April 15, 2026. The detailed search strategies for each database are provided in File S1.

### 2.2 Study Inclusion and Exclusion

Study eligibility was defined according to the PICOS framework. We included studies enrolling adult patients (≥18 years) hospitalized with AHF, including acute decompensated heart failure (ADHF), new‐onset AHF, or worsening chronic heart failure (CHF) requiring urgent admission, regardless of left ventricular ejection fraction (LVEF) phenotype or etiology. The exposure of interest was elevated circulating GDF‐15 levels measured at admission or during the early acute phase, defined as within 48 h of hospital presentation. Patients with lower GDF‐15 levels, based on study‐specific cutoff values (e.g., median, tertiles, quartiles, or predefined thresholds), served as comparators. The primary outcome was all‐cause mortality during follow‐up. Eligible study designs included observational cohort studies (prospective or retrospective) and post‐hoc analyses of randomized controlled trials that reported associations between baseline GDF‐15 levels and mortality risk in this population.

Studies were excluded if they: (1) enrolled patients with stable chronic HF without an acute hospitalization episode, (2) included mixed cardiovascular populations (e.g., acute coronary syndrome or general cardiology cohorts) without separate data for AHF patients, (3) measured GDF‐15 beyond the acute admission window (e.g., only at discharge or during follow‐up rather than within 48 h of admission), (4) did not report all‐cause mortality outcomes or did not provide extractable risk estimates comparing high versus low GDF‐15 categories, (5) were case reports, case series, cross‐sectional studies, reviews, editorials, or non‐human studies, or (6) involved duplicate or overlapping cohorts without the most complete dataset available. When multiple publications were found to originate from the same underlying patient cohort, only the report providing the most comprehensive data or the largest study population was included in order to avoid duplicate counting of participants.

### 2.3 Study Quality Assessment

Two reviewers independently conducted the literature search, screened studies for eligibility, performed quality assessment, and extracted data, with any discrepancies resolved through discussion and consensus among the authors. Methodological quality was evaluated using the Newcastle–Ottawa Scale (NOS) [33], which assesses key domains including cohort selection, comparability through adjustment for confounding factors, and adequacy of outcome ascertainment. The NOS yields a total score ranging from 1 to 9, with higher scores reflecting greater methodological rigor. In this review, studies with scores of ≥8 were considered high quality.

### 2.4 Data Extraction

Extracted data encompassed study‐level characteristics (first author, publication year, study design, and country of origin), as well as detailed patient information including sample size, mean age, sex distribution, the proportion of patients with heart failure with reduced ejection fraction (HFrEF), and the proportion with ischemic etiology. We also collected exposure‐related details, including the timing of blood sampling during admission, the assay methods used to measure circulating GDF‐15, the approaches applied to determine study‐specific cutoff values, and the cutoff values of GDF‐15 used to define high versus low GDF‐15 categories defined at the study level. In addition, follow‐up duration, the number of deaths observed during follow‐up, and the covariates included in multivariable models were extracted to characterize the extent of confounding adjustment in the reported associations between baseline GDF‐15 levels and subsequent mortality risk.

### 2.5 Statistical Analysis

The association between admission circulating GDF‐15 levels and mortality risk in patients with AHF was summarized as risk ratios (RRs) with corresponding 95% confidence intervals (CIs), comparing patients with high versus low baseline GDF‐15 concentrations. When necessary, RRs and standard errors were derived from reported confidence intervals or p‐values and subsequently log‐transformed to stabilize variance and approximate normality [32]. We preferentially extracted the most fully adjusted RRs with corresponding 95% CIs from each study, rather than deriving crude estimates from event counts, to minimize the influence of confounding. Because all included studies reported effect estimates with CIs, no back‐calculation from raw data was required for this meta‐analysis. For meta‐analysis, RRs were log‐transformed and standard errors were derived from the reported CIs, which is the standard approach for variance stabilization and statistical pooling [32]. Between‐study inconsistency was assessed using the Cochrane Q statistic and quantified using the *I*² metric, which provides an estimate of the degree of variability in effect estimates that may reflect underlying heterogeneity across studies [34]. Studies with values of *I*² < 25%, 25%–75%, and > 75% were interpreted as with low, moderate, and high heterogeneity, respectively. Pooled effect estimates were calculated using a random‐effects model to account for anticipated clinical and methodological variability across studies [32]. The robustness of the overall association was examined through leave‐one‐out sensitivity analyses [35], and additional sensitivity analyses were conducted by restricting the synthesis to high‐quality studies (NOS ≥ 8). Prespecified subgroup analyses were performed to explore whether study‐level characteristics influenced the observed associations, including study country (Asian vs. Western countries), study design (prospective vs. retrospective or post‐hoc analyses), mean patient age, timing and assay methods of GDF‐15 measurement, cutoff values defining high GDF‐15, follow‐up duration, analytic models (univariate vs. multivariate adjustment), and whether baseline B‐type natriuretic peptide (BNP) or N‐terminal pro‐BNP (NT‐proBNP) was included as an adjusted covariate. Potential publication bias was evaluated by visual inspection of funnel plot symmetry and Egger's regression test [36]. A two‐sided *p*‐value < 0.05 was considered statistically significant. All statistical analyses were performed using RevMan (version 5.3; Cochrane Collaboration, Oxford, UK) and Stata (version 17.0; StataCorp, College Station, TX, USA).

## 3 Results

### 3.1 Study Inclusion

Figure S1 depicts the study selection workflow. A total of 670 records were retrieved from the 3 databases, of which 156 duplicates were removed. Screening of titles and abstracts resulted in the exclusion of 491 records that did not meet the eligibility criteria. The full texts of the remaining 23 articles were evaluated independently by 2 reviewers, and 13 were excluded for reasons shown in Figure S1. Ultimately, 10 studies met all criteria and were included in the quantitative synthesis [21, 22, 23, 24, 25, 26, 27, 28, 29, 30].

### 3.2 Overview of the Study Characteristics

The key characteristics of the included studies are summarized in Table 1. A total of 10 studies published between 2016 and 2025 were included, comprising 8 prospective cohort studies [22, 23, 25, 26, 27, 28, 29, 30] and 1 retrospective cohort study [24], as well as one large post hoc analysis of a randomized trial cohort [21]. These studies represented diverse geographic regions, including Serbia, China, Portugal, Romania, Thailand, Japan, Spain, and a multinational cohort from Europe and the United States. Sample sizes varied substantially, ranging from 84 to 1391 participants, yielding a combined study population of 3724 patients hospitalized with AHF. The mean age of participants ranged from 61.0 to 74.0 years, and the proportion of men varied from 47.6% to 77.8% across studies. The included cohorts represented a heterogeneous spectrum of HF phenotypes, with seven studies enrolling mixed populations irrespective of LVEF [21, 22, 23, 24, 25, 26, 28], while three focused on specific subgroups such as HFpEF [27, 30] and HFrEF [29]. The proportion of ischemic etiology ranged from 11.6% to 69.8% among these studies. All studies assessed circulating GDF‐15 during the acute admission phase, with blood sampling performed at admission [21, 25, 29] or within 24–48 h of hospitalization [22, 23, 24, 26, 27, 28, 30]. GDF‐15 was measured using enzyme‐linked immunosorbent assay (ELISA) [21, 22, 25, 29], electrochemiluminescence immunoassay (ECLIA) [23, 24, 26, 28, 30], or a Luminex bead‐based multiplex assay [27]. Cutoff definitions for high versus low GDF‐15 differed considerably across studies: five studies [21, 22, 26, 28, 29] used median‐based thresholds, three studies [23, 24, 25] applied ROC curve‐derived cutoffs, and two studies [27, 30] compared tertile categories (T3 vs. T1). Reported cutoff values ranged from 618 to 6222 ng/L. Follow‐up duration varied widely, from 1 to 66 months. Across all included cohorts, a total of 882 deaths were recorded during follow‐up. Multivariate adjustment was performed in eight studies [21, 22, 23, 24, 26, 27, 29, 30], with covariates commonly including age, sex, renal function indices, natriuretic peptides (BNP or NT‐proBNP), cardiac function parameters, comorbidities, and other clinical risk markers, although two smaller cohorts [25, 28] reported unadjusted associations only.

**Table 1:** Characteristics of the included studies.

Study	Country	Design	Sample size	Mean age (years)	Men (%)	HFrEF (%)	Ischemic (%)	Timing for the measurements of blood GDF‐15	Methods for measuring blood GDF‐15	Methods for determine the cutoff of GDF‐15	Cutoff value of GDF‐15 (ng/L)	Follow‐up duration (months)	No. of patients died during follow‐up	Variables adjusted
Jankovic [22]	Serbia	PC	107	70.0	63.6	NR	50.5	Within 24 h of admission	ELISA	Median	3481	12	37	Age, sex, ischemic etiology, LVEF, BNP, SCr, eGFR, Hb, hsCRP, BUN, hsTnI, and fibrinogen
Demissei [21]	The Netherlands, UK, Italy, Poland, and USA	Post‐hoc analysis	1391	70.2	67.1	71.6	69.8	At admission	ELISA	Median	4500	6	358	Age, sex, BMI, vital signs (SBP, DBP, HR, RR), orthopnea, rales, edema, JVP, NYHA class, medical history (AF, COPD, stroke, PVD, HTN, DM, hypercholesterolemia, IHD, prior HF hospitalization), prior medication use (ACEi/ARB, beta‐blocker, CCB, MRA), device (ICD), and study drug
Hao [23]	China	PC	260	61.0	65.0	NR	11.6	Within 24 h of admission	ECLIA	ROC curve analysis	4526	12	46	Age, sex, sodium, RDW, and DM
Lourenco [24]	Portugal	RC	249	74.0	53.8	60.0	55.0	Within 24 h of admission	ECLIA	ROC curve analysis	3500	36	147	Age, sex, NYHA class at admission, SBP at admission, DM, HTN, AF, IHD, discharge BNP, hs‐TnT, CRP, BNP decrease >30% during hospitalization, renal dysfunction, anemia, severe systolic dysfunction, and evidence‐based therapy (BB, ACEi/ARB, MRA)
Miftode [25]	Romania	PC	120	66.4	59.2	NR	49.2	At admission	ELISA	ROC curve analysis	618	1	21	None
Gürgöze [26]	The Netherlands	PC	386	74.0	63.0	83.0	49.0	Within 48 h of admission	ECLIA	Median	4632	10.7	81	Age, sex, systolic BP, diabetes, LVEF, previous HF hospitalization within last 6 months, ischemic HF etiology, BMI, eGFR and NT‐proBNP
Yin [27]	China	PC	380	71.0	49.5	0	53.9	Within 48 h of admission	Luminex bead‐based multiplex assay	T3:T1	4228	12	63	Age, sex, ASCEND‐HF risk score, history of HF, NT‐proBNP, and hs‐TnT
Kosum [28]	Thailand	PC	84	69.0	47.6	23.1	38.1	Within 24 h of admission	ECLIA	Median	6222	7	21	None
Otaki [30]	Japan	PC	643	73.0	58.2	0	35.2	Within 24 h of admission	ECLIA	T3:T1	3340	66	88	Age, sex, NYHA class, DM, AF, CKD, anemia, and NT‐proBNP
Cortés [29]	Spain	PC	104	66.7	77.8	100.0	31.0	At admission	ELISA	Median	3100	23.5	20	Age, sex, eGFR, and sST2

Abbreviations: ACEi, angiotensin‐converting enzyme inhibitor; ADHF, acute decompensated heart failure; AF, atrial fibrillation; AHF, acute heart failure; ARB, angiotensin receptor blocker; ASCEND‐HF, Acute Study of Clinical Effectiveness of Nesiritide in Decompensated Heart Failure; BB, beta‐blocker; BMI, body mass index; BNP, B‐type natriuretic peptide; BUN, blood urea nitrogen; CCB, calcium channel blocker; CKD, chronic kidney disease; COPD, chronic obstructive pulmonary disease; CRP, C‐reactive protein; DBP, diastolic blood pressure; DM, diabetes mellitus; ECLIA, electrochemiluminescence immunoassay; eGFR, estimated glomerular filtration rate; ELISA, enzyme‐linked immunosorbent assay; GDF‐15, growth differentiation factor 15; Hb, hemoglobin; HF, heart failure; HFrEF, heart failure with reduced ejection fraction; HR, heart rate; hsCRP, high‐sensitivity C‐reactive protein; hsTnI, high‐sensitivity troponin I; hs‐TnT, high‐sensitivity troponin T; HTN, hypertension; ICD, implantable cardioverter‐defibrillator; IHD, ischemic heart disease; JVP, jugular venous pressure; LVEF, left ventricular ejection fraction; MRA, mineralocorticoid receptor antagonist; NR, not reported; NT‐proBNP, N‐terminal pro‐B‐type natriuretic peptide; NYHA, New York Heart Association; PC, prospective cohort; PVD, peripheral vascular disease; RC, retrospective cohort; RDW, red blood cell distribution width; ROC, receiver operating characteristic; RR, respiratory rate; SBP, systolic blood pressure; SCr, serum creatinine; sST2, soluble suppression of tumorigenicity 2; T3:T1, tertile 3 versus tertile 1.

### 3.3 Study Quality Evaluation

Study quality was assessed using the NOS, with detailed results presented in Table S1. Total NOS scores ranged from 6 to 9, indicating that the overall methodological quality of the included evidence base was moderate to high. Six studies [22, 23, 24, 27, 29, 30] achieved the highest score of 9, reflecting strong cohort representativeness, clearly defined exposure measurement during admission, appropriate adjustment for confounding (including age and additional clinical variables), standardized mortality assessment, and sufficiently complete follow‐up. One study scored 8, generally demonstrating robust design and outcome assessment but with minor limitations related to short follow‐up duration [26]. One study scored 7 [21] mainly due to limited representativeness of the exposed cohort and an insufficiently long follow‐up duration. Two studies scored 6 [25, 28], primarily due to limited adjustment for confounding factors and shorter or less clearly adequate follow‐up. Overall, no study was judged to be of poor quality, supporting the reliability of the available evidence on admission GDF‐15 as a prognostic biomarker in AHF.

### 3.4 Association between GDF‐15 at Baseline and Mortality Risk

**Figure 1:**
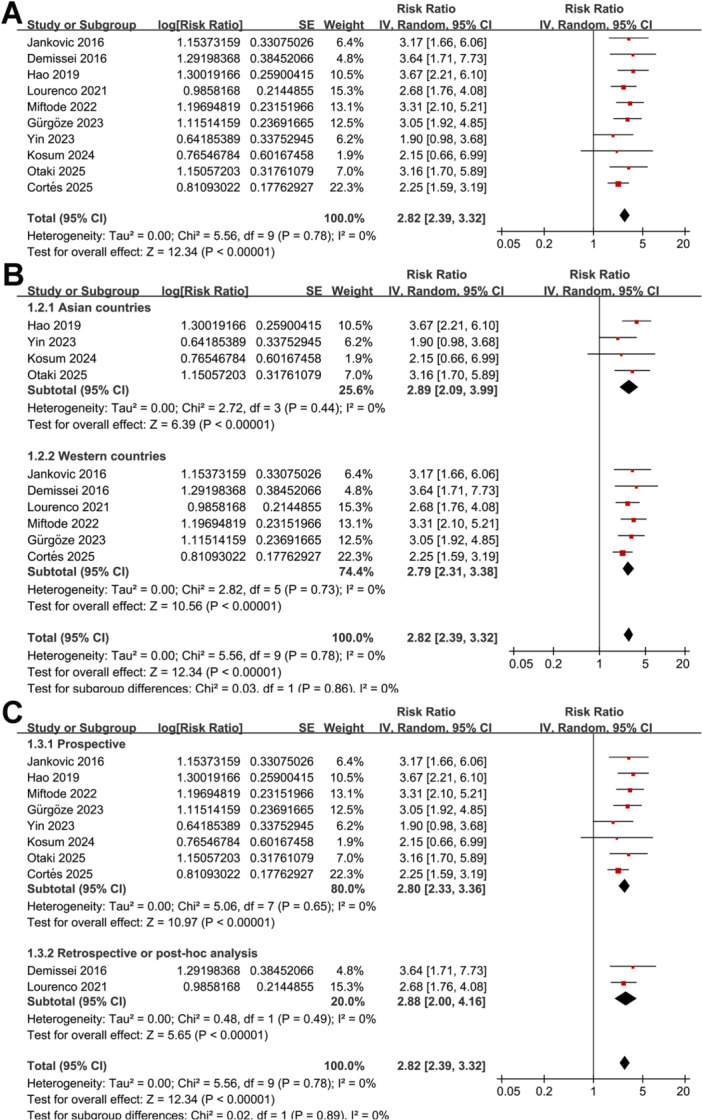
Forest plots for the meta‐analysis of the association between baseline blood level of GDF‐15 and mortality risk of patients with AHF. A, overall meta‐analysis; B, subgroup analysis according to study region; and C, subgroup analysis according to study design.

Pooled results of the 10 studies [21, 22, 23, 24, 25, 26, 27, 28, 29, 30] showed that a higher blood level of GDF‐15 on admission was associated with an increased mortality of patients with AHF during follow‐up (RR: 2.82, 95% CI: 2.39 to 3.32, *p* < 0.001; Figure 1A) with no evidence of between‐study heterogeneity (*p* for Cochrane *Q* test = 0.78; *I* ^2^ = 0%). Sensitivity analysis by excluding one study at a time showed consistent results (RR: 2.73 to 3.00, *p* all < 0.05). In addition, the sensitivity analysis limited to studies with NOS ≥ 8 also showed similar results (RR: 2.72, 95% CI: 2.26 to 3.27, *p* < 0.001; *I* ^2^ = 0%).

**Figure 2:**
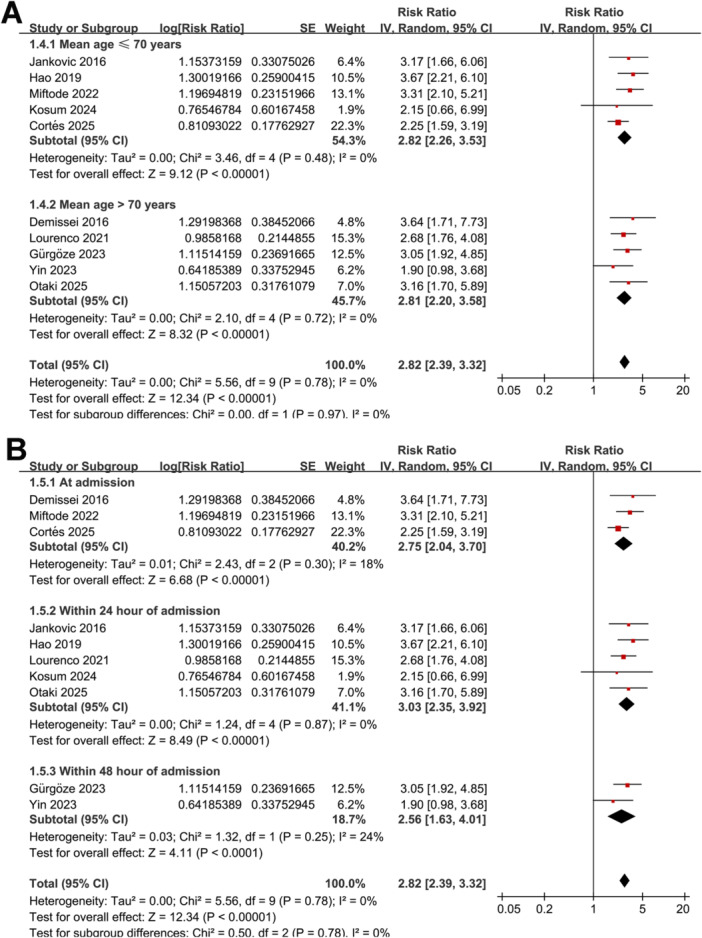
Forest plots for the subgroup analysis of the association between baseline blood level of GDF‐15 and mortality risk of patients with AHF. A, subgroup analysis according to the mean ages of the patients; and B, subgroup analysis according to the timing of GDF‐15 measuring.

**Figure 3:**
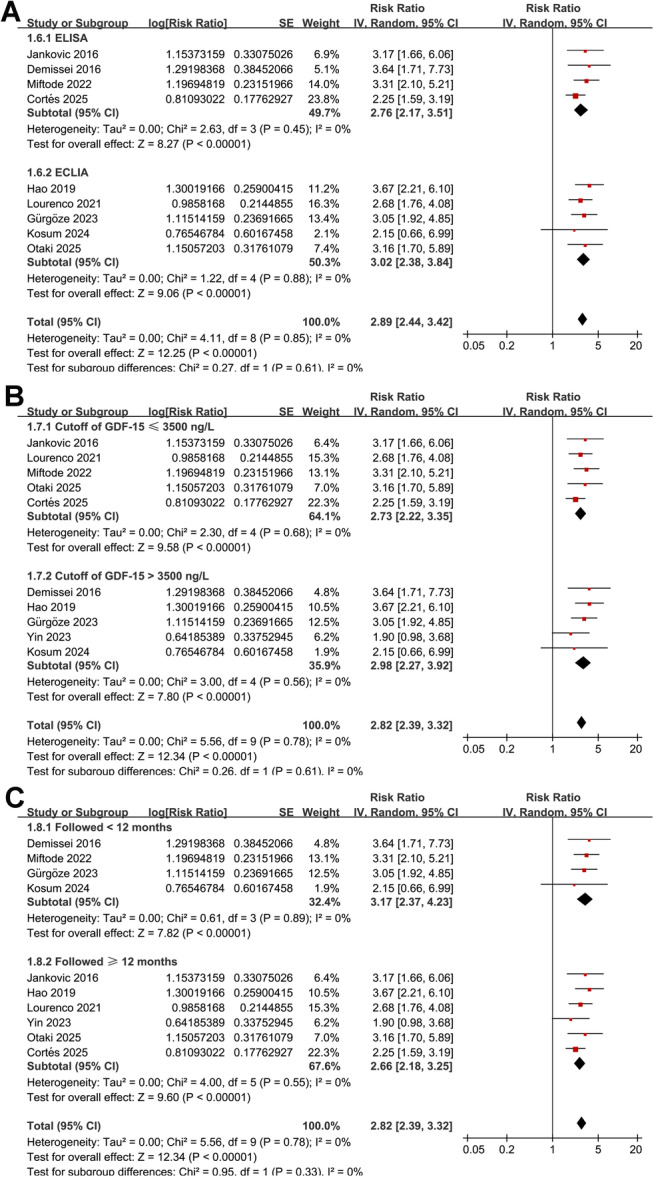
Forest plots for the subgroup analysis of the association between baseline blood level of GDF‐15 and mortality risk of patients with AHF. A, subgroup analysis according to the methods for measuring GDF‐15; B, subgroup analysis according to the cutoff values of GDF‐15; and C, subgroup analysis according to the follow‐up durations.

**Figure 4:**
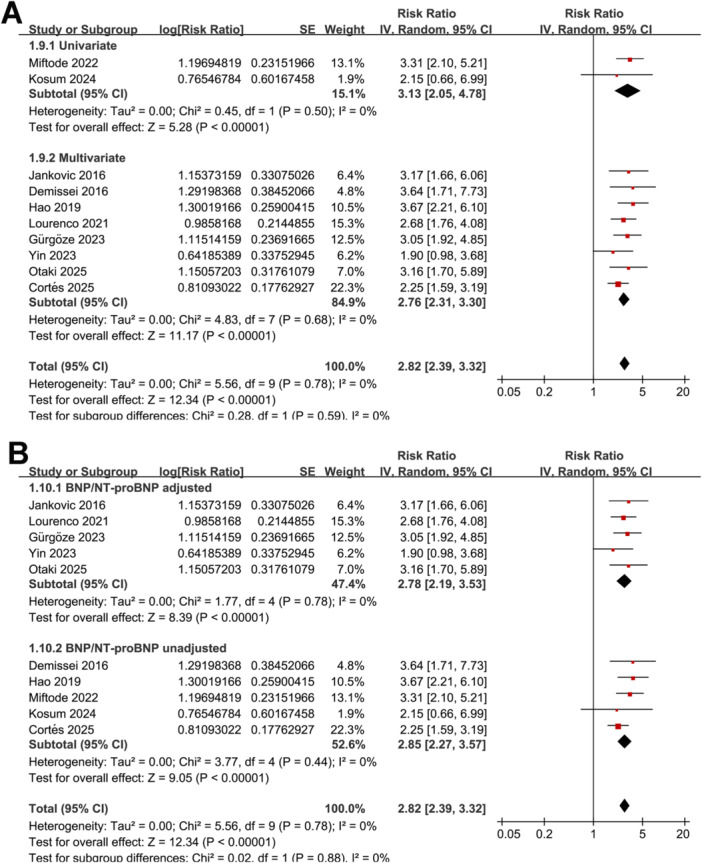
Forest plots for the subgroup analysis of the association between baseline blood level of GDF‐15 and mortality risk of patients with AHF. A, subgroup analysis according to the analytic models; and B, subgroup analysis according to the adjustment of BNP/NT‐proBNP.

Further subgroup analyses showed similar results between studies from Asian and Western countries (RR: 2.89 vs. 2.79, *p* for subgroup difference = 0.86; Figure 1B), in prospective and retrospective or post‐hoc analysis cohorts (RR: 2.80 vs. 2.88, *p* for subgroup difference = 0.89; Figure 1C), in patients with the mean ages ≤ 70 or > 70 years (RR: 2.82 vs. 2.81, *p* for subgroup difference = 0.97; Figure 2A), and among studies with blood level of GDF‐15 measured at admission, and within 24 h or 48 h after admission (RR: 2.75 vs. 3.03 and 2.56, *p* for subgroup difference = 0.78; Figure 2B). Moreover, similar results were observed in studies with blood GDF‐15 measured with ELISA and ECLIA (RR: 2.76 vs. 3.02, *p* for subgroup difference = 0.61; Figure 3A), with the cutoffs of GDF‐15 ≤ 3500 ng/L or >3500 ng/L (RR: 2.73 vs. 2.98, *p* for subgroup difference = 0.61; Figure 3B), and in studies with the follow‐up duration < 12 or ≥ 12 months (RR: 3.17 vs. 2.66, *p* for subgroup difference = 0.33; Figure 3C). Finally, the results were not statistically significant between studies with univariate and multivariate analyses (RR: 3.13 vs. 2.76, *p* for subgroup difference = 0.59; Figure 4A), or between studies with or without adjustment blood BNP/NT‐proBNP level at baseline (RR: 2.78 vs. 2.85, *p* for subgroup difference = 0.88; Figure 4B).

### 3.5 Publication Bias

Figure S2 displays the funnel plots evaluating potential publication bias for the meta‐analyses of the association between blood GDF‐15 level on admission and mortality risk of patients with AHF. No obvious asymmetry was observed on inspection, and Egger's regression tests did not suggest significant small‐study effects (*p* = 0.59).

## 4 Discussion

This meta‐analysis provides a summarized evidence base indicating that elevated circulating GDF‐15 measured at hospital admission (or within the early acute phase) is consistently associated with a higher subsequent risk of all‐cause mortality among patients hospitalized with AHF. Beyond confirming a prognostic signal, the findings suggest that GDF‐15 captures clinically meaningful risk information that is observable early in the hospitalization course and persists across diverse study settings and follow‐up horizons. Because admission is the key decision window for triage, monitoring intensity, and early therapeutic planning, a biomarker that reflects broader systemic stress may be particularly valuable in AHF, where outcomes are shaped not only by hemodynamics but also by multi‐organ dysfunction and inflammatory–metabolic derangements.

Several complementary mechanisms may explain why high GDF‐15 is linked to mortality in AHF. GDF‐15 is a stress‐inducible cytokine within the transforming growth factor‐β superfamily, upregulated by cellular injury, oxidative stress, inflammatory signaling, and tissue hypoxia—processes that are amplified during acute decompensation [37, 38]. From a molecular perspective, experimental work across cardiovascular and systemic injury models has shown that GDF‐15 expression is induced by stress‐response pathways (including inflammatory transcriptional programs and cellular damage signaling), aligning it with the overall severity of acute illness [38]. In HF, higher GDF‐15 likely reflects an integrated burden of myocardial strain and remodeling, endothelial dysfunction, and neurohormonal activation, while simultaneously tracking extra‐cardiac contributors such as renal impairment, hepatic congestion, anemia, and catabolic states [39, 40]. GDF‐15 may also be a marker of frailty‐like biology and metabolic stress [41], which could partly explain its association with adverse prognosis in older and comorbid populations commonly hospitalized with AHF [42]. Importantly, even if GDF‐15 has context‐dependent protective signaling in certain experimental settings (e.g., limiting excessive inflammation) [43], persistently elevated circulating levels in clinical AHF probably indicate a high‐intensity stress response and multi‐organ vulnerability rather than a single causal pathway—consistent with its role as an “integrative” prognostic biomarker [44].

Importantly, accumulating evidence suggests that GDF‐15 may not be solely a passive marker but could also exert direct biological effects. Preclinical data indicate that activation of upstream pathways (e.g., TFEB signaling) can increase circulating GDF‐15 levels and promote adverse cardiac remodeling, while exogenous GDF‐15 has been shown to induce cardiac atrophy and functional impairment in experimental models [45]. These findings raise the possibility that GDF‐15 may participate in the pathophysiology of cardiac dysfunction rather than merely reflecting it [45]. In parallel, recent clinical evidence has demonstrated that pharmacologic inhibition of GDF‐15 using a monoclonal antibody (ponsegromab) results in significant improvements in body weight, appetite, and physical activity in patients with cancer cachexia, thereby establishing GDF‐15 as a therapeutically targetable pathway in humans [46]. Although these findings arise from non‐cardiovascular settings, they provide proof‐of‐concept that modulation of the GDF‐15 axis can produce clinically meaningful effects. Taken together, these data support a more nuanced interpretation in which GDF‐15 may function both as a marker of systemic stress and as a potential mediator of adverse biological processes. However, its mechanistic role in AHF remains incompletely defined, and it is currently unclear whether targeting GDF‐15 would translate into improved cardiovascular outcomes. Therefore, within the context of AHF, GDF‐15 should primarily be regarded as an integrative prognostic biomarker, while its potential as a therapeutic target warrants further investigation.

It should also be noted that GDF‐15 is not specific to HF and may be elevated in a wide range of conditions, including inflammation [38], renal dysfunction [47], malignancy [48], and systemic metabolic stress [11]. As such, its sensitivity and specificity for diagnosing acute decompensation or congestion are limited compared with established biomarkers such as natriuretic peptides. Rather than serving as a diagnostic marker, GDF‐15 appears to reflect the overall burden of systemic stress and multi‐organ dysfunction, which may explain its consistent association with adverse outcomes [11]. Accordingly, its primary clinical utility may lie in prognostic risk stratification rather than in the diagnosis of AHF. On the other hand, AHF encompasses a spectrum of hemodynamic phenotypes, commonly described by the Forrester classification (e.g., “warm–dry,” “warm–wet,” “cold–wet,” and “cold–dry”), which reflect varying degrees of congestion and tissue perfusion [49]. Although the included studies did not provide data stratified by these phenotypes, it is plausible that GDF‐15 levels may differ across these profiles, as the biomarker integrates signals related to systemic inflammation, hypoperfusion, and multi‐organ dysfunction. For example, higher levels might be expected in “cold–wet” patients, who exhibit both congestion and impaired perfusion. However, these considerations remain speculative, and future studies are needed to clarify the role of GDF‐15 across distinct hemodynamic phenotypes in acute heart failure.

The consistency of the association across prespecified subgroup and sensitivity analyses strengthens interpretability. The prognostic relationship remained similar between Asian and Western cohorts and across different study designs (prospective cohorts, retrospective cohort, and a post hoc trial analysis), suggesting that the signal is not confined to a particular healthcare system, recruitment context, or analytic framework. Likewise, broadly comparable estimates were observed across mean age strata, supporting the notion that GDF‐15 reflects adverse biology relevant in both relatively younger and older AHF populations. The association also persisted regardless of whether blood sampling was performed at admission or within 24–48 h, which is reassuring for real‐world implementation because exact sampling time can vary across hospitals while still reflecting the acute‐phase biology. Results were also consistent across measurement platforms (ELISA and ECLIA) and across different cutoff approaches (median, ROC‐derived thresholds, tertile contrasts). This pattern suggests that the prognostic information carried by GDF‐15 is not simply an artifact of a particular assay or dichotomization rule. Nevertheless, the absence of detectable heterogeneity should be interpreted carefully: uniform direction and effect sizes may reflect a truly consistent association, but they may also be influenced by the limited number of studies and the fact that subgroup analyses can be underpowered to detect small between‐group differences.

Sensitivity analyses further support robustness. The leave‐one‐out analysis did not materially change the overall inference, indicating that no single cohort dominated the pooled association. Similarly, restricting the synthesis to high‐quality studies (NOS ≥ 8) yielded comparable results, which reduces concern that the main finding is driven by lower‐quality evidence. Of note, the association remained similar in studies reporting multivariable‐adjusted estimates and in analyses stratified by whether BNP/NT‐proBNP was included as a covariate. This is clinically important because natriuretic peptides are central to AHF assessment; persistence of the GDF‐15 signal despite natriuretic peptide adjustment suggests that GDF‐15 may capture risk dimensions not fully represented by hemodynamic stress biomarkers alone (e.g., systemic inflammation, oxidative injury, renal dysfunction, and global illness severity) [50]. At the same time, residual confounding remains plausible, particularly because covariate sets varied across studies and some cohorts reported unadjusted associations.

This meta‐analysis has several strengths. The literature search was comprehensive and up to date, spanning major databases from inception through April 2026 with supplementary reference screening. The included evidence base consisted predominantly of cohort designs with admission (early‐phase) biomarker measurement, aligning closely with the intended clinical use case. The analysis incorporated multiple prespecified subgroup and sensitivity assessments, which collectively demonstrated stability of the prognostic association across key study characteristics. Additionally, the overall body of evidence showed little statistical inconsistency, and formal evaluation did not suggest major small‐study effects, supporting the credibility of the pooled inference. However, important limitations should also be acknowledged. First, clinical heterogeneity across cohorts was inevitable: patient demographics, comorbidity profiles, HF phenotype (including LVEF distributions), and underlying etiology varied substantially. These differences could influence both baseline GDF‐15 levels and mortality risk. Furthermore, detailed subgroup analyses according to clinically relevant characteristics—such as sex, HF etiology, cardiogenic shock, cardiorenal syndrome, and baseline LVEF—could not be performed because these data were not consistently reported across studies, and individual participant data (IPD) were unavailable. Future studies with standardized reporting or IPD meta‐analyses are warranted to evaluate whether the prognostic value of GDF‐15 varies across these important clinical subgroups. Second, exposure definitions differed in cutoff selection (median, ROC‐based, tertiles), and absolute thresholds varied widely, which limits immediate translation into a single clinically actionable cut point. Third, confounding adjustment was inconsistent across the included studies, both in terms of the type and number of covariates included in multivariable models. Because this meta‐analysis was based on study‐level data, we were unable to directly evaluate the independent contributions of specific covariates or to standardize adjustment strategies across studies. As a result, residual confounding cannot be excluded, and differences in model specification may partly influence the observed association between GDF‐15 and mortality. Nevertheless, the direction and magnitude of the association were broadly consistent across studies with varying adjustment approaches, including those incorporating key clinical variables such as natriuretic peptides and renal function indices, which provides some reassurance regarding the robustness of the findings. Fourth, as an observational synthesis, this meta‐analysis cannot establish causality. Elevated GDF‐15 should be viewed as a prognostic marker reflecting adverse biology rather than proof of a causal mediator of death. Finally, although the lack of statistical heterogeneity is reassuring, the number of included studies remains modest for certain subgroup comparisons, and publication bias assessments have limited power in small meta‐analyses.

Clinically, these findings support admission GDF‐15 as a potential risk‐stratification biomarker in AHF. However, its practical value must be considered in the context of existing clinical assessment and established biomarkers. Unlike natriuretic peptides or cardiac troponins, which are linked to specific pathophysiological processes and clinical decisions (e.g., congestion assessment or myocardial injury), GDF‐15 is a non‐specific marker reflecting integrated systemic stress and multi‐organ dysfunction. As such, an elevated GDF‐15 level may identify patients with heightened vulnerability but does not directly indicate the underlying driver of risk or a specific therapeutic target. This represents an inherent trade‐off: broader prognostic sensitivity at the expense of limited decision specificity. From a pragmatic perspective, the most plausible role of GDF‐15 may lie in complementing, rather than replacing, existing tools. For example, early measurement at admission could help identify patients at high overall risk who may benefit from intensified monitoring, more comprehensive multidisciplinary evaluation (e.g., assessment of renal function, nutritional status, and frailty), or closer post‐discharge follow‐up planning. In this context, GDF‐15 may function as a “global risk signal” that prompts heightened clinical attention rather than directing a specific intervention. However, it remains uncertain whether GDF‐15 provides incremental clinical value beyond routine bedside assessment, standard laboratory parameters, and established biomarkers such as BNP/NT‐proBNP and troponins. Therefore, its integration into clinical practice will depend on demonstrating improvement in risk discrimination, calibration, and reclassification when added to existing models, as well as identifying specific decision points at which its measurement meaningfully alters management. Until such evidence is available, GDF‐15 should be interpreted cautiously and regarded as a complementary prognostic indicator rather than a standalone decision‐making tool. Future research should prioritize (1) IPD meta‐analyses to assess effect modification by LVEF phenotype, etiology, renal function, and treatment patterns; (2) standardization of assays and reporting units with clinically meaningful thresholds; (3) evaluation of serial changes in GDF‐15 during hospitalization and post‐discharge to determine whether trajectories outperform single measurements; and (4) prospective studies testing biomarker‐guided care pathways to establish clinical utility rather than prognostic association alone.

## 5 Conclusions

In conclusion, the current evidence indicates that elevated circulating GDF‐15 measured at admission is a strong and consistent predictor of all‐cause mortality in patients hospitalized with AHF. While causality cannot be inferred and clinical thresholds require further refinement, GDF‐15 appears to capture integrated systemic risk and may serve as a useful adjunct for early risk stratification and future multimarker strategies in AHF.

## Author Contributions

Pingkui Jin and Qinghou Zheng designed the study. Pingkui Jin, Yanjie Geng, Erwei Huo, and Yanhong Xue performed database search, literature review, study quality evaluation, and data extraction. Pingkui Jin, Daofeng You, and Qinghou Zheng performed statistical analyses, and interpreted the results. Pingkui Jin drafted the manuscript. All authors revised the manuscript and approved the submission.

## Conflicts of Interest

The authors declare no conflicts of interest.

## Supporting information


**Figure S1:** Flowchart of database search and study inclusion.

**Figure S2:** Funnel plots estimating the potential publication bias underlying the meta‐analysis of the association between baseline blood level of GDF‐15 and mortality risk of patients with AHF.

**Table S1:** Study quality evaluation via the Newcastle‐Ottawa Scale.

**Supporting File 1:** Detailed search strategy for each database.

## Data Availability

The data that support the findings of this study are available from the corresponding author upon reasonable request.

## References

[1] L. P. Palaniappan , N. B. Allen , Z. I. Almarzooq , et al., “2026 Heart Disease and Stroke Statistics: A Report of US and Global Data From the American Heart Association,” Circulation 153 (2026): e275–e906. 41562125 10.1161/CIR.0000000000001412

[2] T. A. McDonagh , M. Metra , M. Adamo , et al., “2023 Focused Update of the 2021 ESC Guidelines for the Diagnosis and Treatment of Acute and Chronic Heart Failure,” European Heart Journal 44, no. 37 (2023): 3627–3639. 37622666 10.1093/eurheartj/ehad195

[3] S. Kurmani and I. Squire , “Acute Heart Failure: Definition, Classification and Epidemiology,” Current Heart Failure Reports 14, no. 5 (2017): 385–392. 28785969 10.1007/s11897-017-0351-y PMC5597697

[4] S. J. Greene , J. Bauersachs , J. J. Brugts , et al., “Worsening Heart Failure: Nomenclature, Epidemiology, and Future Directions,” Journal of the American College of Cardiology 81, no. 4 (2023): 413–424. 36697141 10.1016/j.jacc.2022.11.023

[5] M. A. Bazmpani , C. A. Papanastasiou , V. Kamperidis , et al., “Contemporary Data on the Status and Medical Management of Acute Heart Failure,” Current Cardiology Reports 24, no. 12 (2022): 2009–2022. 36385324 10.1007/s11886-022-01822-1 PMC9747828

[6] S. Skoularigkis , C. Kourek , A. Xanthopoulos , et al., “Prognostic Models in Heart Failure: Hope or Hype?,” Journal of Personalized Medicine 15, no. 8 (2025): 345. 40863407 10.3390/jpm15080345 PMC12387128

[7] M. Boesing , J. Suchina , G. Lüthi‐Corridori , F. Jaun , M. Brändle , and J. D. Leuppi , “The Early Prediction of Patient Outcomes in Acute Heart Failure: A Retrospective Study,” Journal of Cardiovascular Development and Disease 12, no. 7 (2025): 236. 40710762 10.3390/jcdd12070236 PMC12295718

[8] A. P. Sindone , J. Amerena , C. G. De Pasquale , et al., “Consensus Statement on Optimisation of Patient Care After Hospitalisation for Acute Heart Failure,” Heart, Lung and Circulation 35, no. 2 (2026): 157–170. 10.1016/j.hlc.2025.09.019 41539881

[9] M. Al‐Sadawi , M. Saad , P. Ayyadurai , N. N. Shah , M. Bhandari , and T. J. Vittorio , “Biomarkers in Acute Heart Failure Syndromes: An Update,” Current Cardiology Reviews 18, no. 3 (2022): e090921196330. 34503430 10.2174/1573403X17666210909170415 PMC9615213

[10] N. P. E. Kadoglou , J. Parissis , A. Karavidas , I. Kanonidis , and M. Trivella , “Assessment of Acute Heart Failure Prognosis: The Promising Role of Prognostic Models and Biomarkers,” Heart Failure Reviews 27, no. 2 (2022): 655–663. 34036472 10.1007/s10741-021-10122-9

[11] A. M. di Candia , D. X. de Avila , G. R. Moreira , H. Villacorta , and A. S. Maisel , “Growth Differentiation factor‐15, a Novel Systemic Biomarker of Oxidative Stress, Inflammation, and Cellular Aging: Potential Role in Cardiovascular Diseases,” American Heart Journal Plus: Cardiology Research and Practice 9 (2021): 100046. 38559370 10.1016/j.ahjo.2021.100046 PMC10978141

[12] R. E. Alexa , A. Ceasovschih , B. C. Morărașu , et al., “Growth Differentiation Factor 15 as a Link Between Obesity, Subclinical Atherosclerosis, and Heart Failure: A Systematic Review,” Medicina 62, no. 1 (2026): 132. 41597417 10.3390/medicina62010132 PMC12843032

[13] T. Tian , M. Liu , P. J. Little , H. Strijdom , J. Weng , and S. Xu , “Emerging Roles of GDF15 in Metabolic and Cardiovascular Diseases,” Research (Washington, D.C.) 8 (2025): 0832. 40837873 10.34133/research.0832 PMC12361751

[14] I. Dakota , M. A. Wijayanto , A. S. D. Nugrahani , et al., “Diagnostic and Prognostic Implications of Growth Differentiation Factor 15 in Heart Failure With Preserved Ejection Fraction: A Systematic Review and Meta‐Analysis,” PeerJ 13 (2025): e20168. 41180477 10.7717/peerj.20168 PMC12577569

[15] Y. Wang , C. Zhen , R. Wang , and G. Wang , “Growth‐Differentiation Factor‐15 Predicts Adverse Cardiac Events in Patients With Acute Coronary Syndrome: A Meta‐Analysis,” American Journal of Emergency Medicine 37, no. 7 (2019): 1346–1352. 31029521 10.1016/j.ajem.2019.04.035

[16] O. Afzal , M. Afzal , N. H. Khan , et al., “GDF‐15 as an Integrative Cardiometabolic Biomarker,” Clinica Chimica Acta 583 (2026): 120839. 10.1016/j.cca.2026.120839 41539642

[17] K. Tiwari , A. Saravanan , A. Anil , et al., “Molecular and Functional Significance of Growth Differentiation Factor‐15: A Review on Cardiovascular‐Kidney‐Metabolic Biomarker,” Current Cardiology Reviews 21, no. 3 (2025): e1573403X1332671. 10.2174/011573403X332671241121063641 PMC12172221 39781722

[18] J. W. Luo , W. H. Duan , L. Song , Y. Q. Yu , and D. Z. Shi , “A Meta‐Analysis of Growth Differentiation Factor‐15 and Prognosis in Chronic Heart Failure,” Frontiers in Cardiovascular Medicine 8 (2021): 630818. 34805295 10.3389/fcvm.2021.630818 PMC8602355

[19] L. Lyu , J. Xu , C. Xv , et al., “Prognostic Value of Growth Differentiation Factor‐15 in Heart Failure Among Whole Ejection Fraction Phenotypes,” ESC Heart Failure 11, no. 4 (2024): 2295–2304. 38641904 10.1002/ehf2.14807 PMC11287306

[20] V. Castiglione , A. Aimo , G. Vergaro , L. Saccaro , C. Passino , and M. Emdin , “Biomarkers for the Diagnosis and Management of Heart Failure,” Heart Failure Reviews 27, no. 2 (2022): 625–643. 33852110 10.1007/s10741-021-10105-w PMC8898236

[21] B. G. Demissei , M. A. E. Valente , J. G. Cleland , et al., “Optimizing Clinical Use of Biomarkers in High‐Risk Acute Heart Failure Patients,” European Journal of Heart Failure 18, no. 3 (2016): 269–280. 26634889 10.1002/ejhf.443

[22] R. Jankovic‐Tomasevic , S. U. Pavlovic , T. Jevtovic‐Stoimenov , et al., “Prognostic Utility of Biomarker Growth Differentiation Factor‐ 15 in Patients With Acute Decompensated Heart Failure,” Acta Cardiologica 71, no. 5 (2016): 587–595. 27695017 10.2143/AC.71.5.3167503

[23] J. Hao , I. Cheang , L. Zhang , et al., “Growth Differentiation Factor‐15 Combined With N‐Terminal Prohormone of Brain Natriuretic Peptide Increase 1‐year Prognosis Prediction Value for Patients With Acute Heart Failure: A Prospective Cohort Study,” Chinese Medical Journal 132, no. 19 (2019): 2278–2285. 31567379 10.1097/CM9.0000000000000449 PMC6819038

[24] P. Lourenço , F. M. Cunha , J. Ferreira‐Coimbra , I. Barroso , J. T. Guimarães , and P. Bettencourt , “Dynamics of Growth Differentiation Factor 15 in Acute Heart Failure,” ESC Heart Failure 8, no. 4 (2021): 2527–2534. 33938154 10.1002/ehf2.13377 PMC8318469

[25] R. S. Miftode , D. Constantinescu , C. M. Cianga , et al., “A Rising Star of the Multimarker Panel: Growth Differentiation Factor‐15 Levels Are an Independent Predictor of Mortality in Acute Heart Failure Patients Admitted to an Emergency Clinical Hospital From Eastern Europe,” Life 12, no. 12 (2022): 1948. 36556311 10.3390/life12121948 PMC9784402

[26] M. T. Gürgöze , L. C. van Vark , S. J. Baart , et al., “Multimarker Analysis of Serially Measured GDF‐15, NT‐proBNP, ST2, GAL‐3, cTni, Creatinine, and Prognosis in Acute Heart Failure,” Circulation: Heart Failure 16, no. 1 (2023): e009526. 36408685 10.1161/CIRCHEARTFAILURE.122.009526 PMC9833118

[27] D. Yin , X. Yan , X. Bai , A. Tian , Y. Gao , and J. Li , “Prognostic Value of Growth Differentiation Factors 15 in Acute Heart Failure Patients With Preserved Ejection Fraction,” ESC Heart Failure 10, no. 2 (2023): 1025–1034. 36519216 10.1002/ehf2.14271 PMC10053169

[28] P. Kosum , N. Siranart , N. Mattanapojanat , et al., “GDF‐15: A Novel Biomarker of Heart Failure Predicts Short‐Term and Long‐Term Heart‐Failure Rehospitalization and Short‐Term Mortality in Patients With Acute Heart Failure Syndrome,” BMC Cardiovascular Disorders 24, no. 1 (2024): 151. 38475710 10.1186/s12872-024-03802-5 PMC10936070

[29] M. Cortés , J. Lumpuy‐Castillo , C. S. García‐Talavera , et al., “New Biomarkers in the Prognostic Assessment of Acute Heart Failure With Reduced Ejection Fraction: Beyond Natriuretic Peptides,” International Journal of Molecular Sciences 26, no. 3 (2025): 986. 39940753 10.3390/ijms26030986 PMC11817831

[30] Y. Otaki , T. Watanabe , M. Shimizu , et al., “Growth Differentiation factor‐15 and N‐Terminal pro‐BNP in Acute Heart Failure With Preserved Ejection Fraction,” ESC Heart Failure 12, no. 2 (2025): 888–899. 39899430 10.1002/ehf2.15068 PMC11911576

[31] M. J. Page , J. E. McKenzie , P. M. Bossuyt , et al., “The PRISMA 2020 Statement: An Updated Guideline for Reporting Systematic Reviews,” BMJ 372 (2021): n71. 33782057 10.1136/bmj.n71 PMC8005924

[32] J. Higgins , J. Thomas , J. Chandler , et al., Cochrane Handbook for Systematic Reviews of Interventions Version 6.2. The Cochrane Collaboration, 2021.

[33] G. A. Wells , B. Shea , D. O'Connell , et al. The Newcastle‐Ottawa Scale (NOS) for Assessing the Quality of Nonrandomised Studies in Meta‐analyses. 2010, http://www.ohri.ca/programs/clinical_epidemiology/oxford.asp.

[34] J. P. T. Higgins and S. G. Thompson , “Quantifying Heterogeneity in a Meta‐Analysis,” Statistics in Medicine 21, no. 11 (2002): 1539–1558. 12111919 10.1002/sim.1186

[35] M. F. Marušić , M. Fidahić , C. M. Cepeha , L. G. Farcaș , A. Tseke , and L. Puljak , “Methodological Tools and Sensitivity Analysis for Assessing Quality or Risk of Bias Used in Systematic Reviews Published in the High‐Impact Anesthesiology Journals,” BMC Medical Research Methodology 20, no. 1 (2020): 121. 32423382 10.1186/s12874-020-00966-4 PMC7236513

[36] M. Egger , G. D. Smith , M. Schneider , and C. Minder , “Bias in Meta‐Analysis Detected by a Simple, Graphical Test,” BMJ 315, no. 7109 (1997): 629–634. 9310563 10.1136/bmj.315.7109.629 PMC2127453

[37] M. Asrih , S. Wei , T. T. Nguyen , H. S. Yi , D. Ryu , and K. Gariani , “Overview of Growth Differentiation Factor 15 in Metabolic Syndrome,” Journal of Cellular and Molecular Medicine 27, no. 9 (2023): 1157–1167. 36992609 10.1111/jcmm.17725 PMC10148061

[38] A. Schwarz , R. Kinscherf , and G. A. Bonaterra , “Role of the Stress‐ and Inflammation‐Induced Cytokine GDF‐15 in Cardiovascular Diseases: From Basic Research to Clinical Relevance,” Reviews in Cardiovascular Medicine 24, no. 3 (2023): 81. 39077481 10.31083/j.rcm2403081 PMC11264000

[39] C. Lombardi , M. Marandola , V. Loria , A. Urbani , and S. Baroni , “Growth Differentiation Factor‐15 as an Emerging Biomarker in Cardiology: Diagnostic and Prognostic Implications,” Journal of Personalized Medicine 16, no. 1 (2026): 16. 41590509 10.3390/jpm16010016 PMC12843272

[40] A. A. Manolis , T. A. Manolis , and A. S. Manolis , “Neurohumoral Activation in Heart Failure,” International Journal of Molecular Sciences 24, no. 20 (2023): 15472. 37895150 10.3390/ijms242015472 PMC10607846

[41] J. L. Sánchez‐Sánchez , Y. Rolland , A. Lucas , S. Guyonnet , B. Vellas , and P. de Souto Barreto , “Associations Between Growth Differentiating Factor‐15 and Frailty in Older Adults From the MAPT Study,” Journal of Cachexia, Sarcopenia and Muscle 17, no. 1 (2026): e70182. 41560403 10.1002/jcsm.70182 PMC12820345

[42] S. Lian , Y. Rolland , and P. de Souto Barreto, , “Growth Differentiation Factor 15 as a Biomarker of Frailty: Evidence From Recent Studies,” Current Opinion in Clinical Nutrition and Metabolic Care 29 (2026): 214–223. 41607211 10.1097/MCO.0000000000001213

[43] H. H. Luan , A. Wang , B. K. Hilliard , et al., “GDF15 Is an Inflammation‐Induced Central Mediator of Tissue Tolerance,” Cell 178, no. 5 (2019): 1231–1244.e11. 31402172 10.1016/j.cell.2019.07.033 PMC6863354

[44] D. Ceelen , A. A. Voors , J. Tromp , et al., “Pathophysiological Pathways Related to High Plasma Growth Differentiation Factor 15 Concentrations in Patients With Heart Failure,” European Journal of Heart Failure 24, no. 2 (2022): 308–320. 34989084 10.1002/ejhf.2424 PMC9302623

[45] M. Ozcan , Z. Guo , C. Valenzuela Ripoll , et al., “Sustained Alternate‐Day Fasting Potentiates Doxorubicin Cardiotoxicity,” Cell Metabolism 35, no. 6 (2023): 928–942.e4. 36868222 10.1016/j.cmet.2023.02.006 PMC10257771

[46] J. D. Groarke , J. Crawford , S. M. Collins , et al., “Ponsegromab for the Treatment of Cancer Cachexia,” New England Journal of Medicine 391, no. 24 (2024): 2291–2303. 39282907 10.1056/NEJMoa2409515

[47] C. Delrue , R. Speeckaert , J. R. Delanghe , and M. M. Speeckaert , “Growth Differentiation Factor 15 (GDF‐15) in Kidney Diseases,” Advances in Clinical Chemistry 114 (2023): 1–46. 37268330 10.1016/bs.acc.2023.02.003

[48] L. Fang , F. Li , and C. Gu , “GDF‐15: A Multifunctional Modulator and Potential Therapeutic Target in Cancer,” Current Pharmaceutical Design 25, no. 6 (2019): 654–662. 30947652 10.2174/1381612825666190402101143

[49] O. Chioncel , A. Mebazaa , A. P. Maggioni , et al., “Acute Heart Failure Congestion and Perfusion Status—Impact of the Clinical Classification on In‐Hospital and Long‐Term Outcomes; Insights from the ESC‐EORP‐HFA Heart Failure Long‐Term Registry,” European Journal of Heart Failure 21, no. 11 (2019): 1338–1352. 31127678 10.1002/ejhf.1492

[50] P. L. Myhre , C. Prebensen , H. Strand , et al., “Growth Differentiation Factor 15 Provides Prognostic Information Superior to Established Cardiovascular and Inflammatory Biomarkers in Unselected Patients Hospitalized With COVID‐19,” Circulation 142, no. 22 (2020): 2128–2137. 33058695 10.1161/CIRCULATIONAHA.120.050360 PMC7688084

