# Supplementary figures and images for: Circulating Level of Growth‐Differentiation Factor 15 and Mortality of Patients With Acute Heart Failure: A Meta‐Analysis
Source: Clin Cardiol. 2026 May 6;49(5):e70338. doi: 10.1002/clc.70338 (PMC13147355; doi:10.1002/clc.70338)

**Supplemental Figure 1** Flowchart of database search and study inclusion.


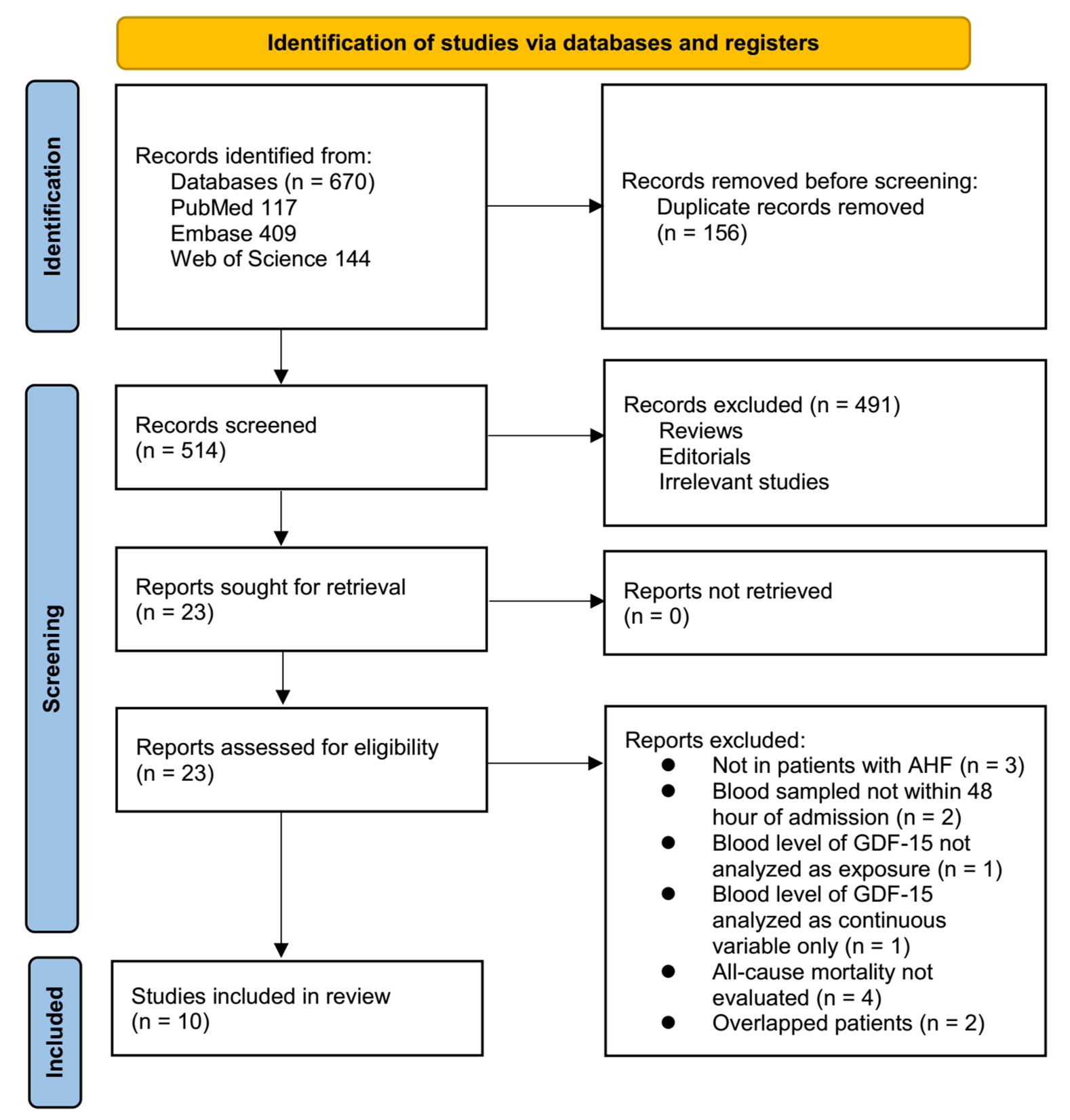

Supplement: Supplementary file 1 — Figure S1: Flowchart of database search and study inclusion. [file CLC-49-e70338-s003.docx]
