# Supplementary material for: Circulating Level of Growth‐Differentiation Factor 15 and Mortality of Patients With Acute Heart Failure: A Meta‐Analysis
Source: Clin Cardiol. 2026 May 6;49(5):e70338. doi: 10.1002/clc.70338 (PMC13147355; doi:10.1002/clc.70338)

**Supplemental Figure 2** Funnel plots estimating the potential publication bias underlying the meta-analysis of the association between baseline blood level of GDF-15 and mortality risk of patients with AHF.


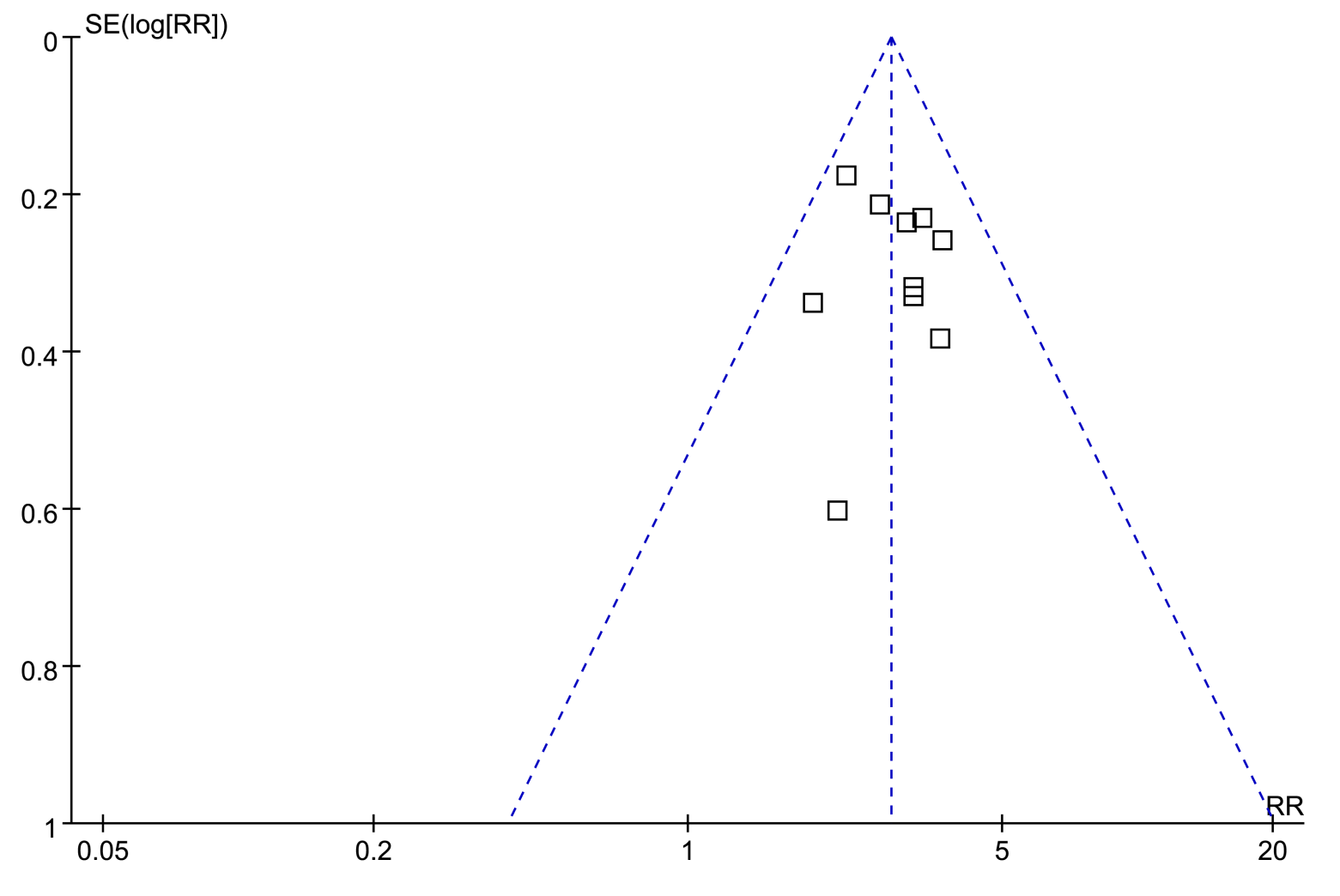

Supplement: Supplementary file 2 — Figure S2: Funnel plots estimating the potential publication bias underlying the meta‐analysis of the association between baseline blood level of GDF‐15 and mortality risk of patients with AHF. [file CLC-49-e70338-s002.docx]
