# Supplementary material for: Circulating Level of Growth‐Differentiation Factor 15 and Mortality of Patients With Acute Heart Failure: A Meta‐Analysis
Source: Clin Cardiol. 2026 May 6;49(5):e70338. doi: 10.1002/clc.70338 (PMC13147355; doi:10.1002/clc.70338)
Supplement: Supplementary file 3 — Table S1: Study quality evaluation via the Newcastle‐Ottawa Scale. [file CLC-49-e70338-s004.docx]

**Supplemental File 1** Detailed search strategy for each database

**PubMed**

("Growth Differentiation Factor 15"[tiab] OR "growth differentiation factor-15"[tiab] OR "GDF-15"[tiab] OR "GDF 15"[tiab] OR "macrophage inhibitory cytokine 1"[tiab] OR "MIC-1"[tiab] OR "prostate differentiation factor"[tiab]) AND ("Heart Failure"[Mesh] OR "heart failure"[tiab] OR "cardiac failure"[tiab] OR "cardiac dysfunction"[tiab] OR "cardiac insufficiency"[tiab]) AND (acute[tiab] OR acutely[tiab] OR decompensat*[tiab] OR decompensation[tiab] OR decompensated[tiab] OR "acute heart failure"[tiab] OR "acute decompensated heart failure"[tiab] OR "worsening heart failure"[tiab]) AND ("Mortality"[Mesh] OR "Survival"[Mesh] OR "Prognosis"[Mesh] OR mortality[tiab] OR death[tiab] OR deaths[tiab] OR survival[tiab] OR outcome*[tiab] OR prognos*[tiab] OR prognostic*[tiab] OR cohort[tiab] OR longitudinal[tiab] OR prospective[tiab] OR retrospect*[tiab] OR followed[tiab] OR "follow-up"[tiab] OR follow[tiab])

**Embase**

1. (growth differentiation factor 15 or growth differentiation factor-15 or gdf-15 or gdf 15 or macrophage inhibitory cytokine 1 or mic-1 or prostate differentiation factor).ti,ab,kw.

2. heart failure/ or (heart failure or cardiac failure or cardiac dysfunction or cardiac insufficiency).ti,ab,kw.

3. (acute or acutely or decompensat* or "acute decompensated" or "acute heart failure" or "worsening heart failure").ti,ab,kw.

4. mortality/ or survival/ or prognosis/ or (mortality or death or deaths or survival or outcome* or prognos* or prognostic* or cohort or longitudinal or prospective or retrospect* or followed or follow-up or follow).ti,ab,kw.

5. 1 and 2 and 3 and 4

**Web of Science**

TS=(("growth differentiation factor 15" OR "growth differentiation factor-15" OR "GDF-15" OR "GDF 15" OR "macrophage inhibitory cytokine 1" OR "MIC-1" OR "prostate differentiation factor") AND ("heart failure" OR "cardiac failure" OR "cardiac dysfunction" OR "cardiac insufficiency") AND (acute OR acutely OR decompensat* OR "acute decompensated" OR "decompensation" OR "decompensated") AND (mortality OR death OR deaths OR survival OR outcome* OR prognos* OR prognostic* OR cohort OR longitudinal OR prospective OR prospectively OR retrospective OR retrospectively OR followed OR "follow-up" OR follow))
