# Supplementary material for: Circulating Level of Growth‐Differentiation Factor 15 and Mortality of Patients With Acute Heart Failure: A Meta‐Analysis
Source: Clin Cardiol. 2026 May 6;49(5):e70338. doi: 10.1002/clc.70338 (PMC13147355; doi:10.1002/clc.70338)
Supplement: Supplementary file 4 — Supporting File 1: Detailed search strategy for each database. [file CLC-49-e70338-s001.docx]

Supplemental Table 1 Study quality evaluation via the Newcastle-Ottawa Scale

| Study | Representativeness of the exposed cohort | Selection of the non-exposed cohort | Ascertainment of exposure | Outcome not present at baseline | Control for age | Control for other confounding factors | Assessment of outcome | Enough long follow-up duration | Adequacy of follow-up of cohorts | Total |
| --- | --- | --- | --- | --- | --- | --- | --- | --- | --- | --- |
| Jankovic 2016 | 1 | 1 | 1 | 1 | 1 | 1 | 1 | 1 | 1 | 9 |
| Demissei 2016 | 0 | 1 | 1 | 1 | 1 | 1 | 1 | 0 | 1 | 7 |
| Hao 2019 | 1 | 1 | 1 | 1 | 1 | 1 | 1 | 1 | 1 | 9 |
| Lourenco 2021 | 1 | 1 | 1 | 1 | 1 | 1 | 1 | 1 | 1 | 9 |
| Miftode 2022 | 1 | 1 | 1 | 1 | 0 | 0 | 1 | 0 | 1 | 6 |
| Gürgöze 2023 | 1 | 1 | 1 | 1 | 1 | 1 | 1 | 0 | 1 | 8 |
| Yin 2023 | 1 | 1 | 1 | 1 | 1 | 1 | 1 | 1 | 1 | 9 |
| Kosum 2024 | 1 | 1 | 1 | 1 | 0 | 0 | 1 | 0 | 1 | 6 |
| Otaki 2025 | 1 | 1 | 1 | 1 | 1 | 1 | 1 | 1 | 1 | 9 |
| Cortés 2025 | 1 | 1 | 1 | 1 | 1 | 1 | 1 | 1 | 1 | 9 |
